# Endurance Exercise Mobilizes Developmentally Early Stem Cells into Peripheral Blood and Increases Their Number in Bone Marrow: Implications for Tissue Regeneration

**DOI:** 10.1155/2016/5756901

**Published:** 2015-11-09

**Authors:** Krzysztof Marycz, Katarzyna Mierzejewska, Agnieszka Śmieszek, Ewa Suszynska, Iwona Malicka, Magda Kucia, Mariusz Z. Ratajczak

**Affiliations:** ^1^Faculty of Biology, University of Environmental and Life Sciences, 50-375 Wroclaw, Poland; ^2^Wroclaw Research Center EIT+, 54-066 Wroclaw, Poland; ^3^Department of Regenerative Medicine, Medical University of Warsaw, 02-091 Warsaw, Poland; ^4^Department of Physiotherapy, University School of Physical Education, 51-617 Wroclaw, Poland; ^5^Stem Cell Biology Program, James Graham Brown Cancer Center, Louisville, KY 40202, USA

## Abstract

Endurance exercise has been reported to increase the number of circulating hematopoietic stem/progenitor cells (HSPCs) in peripheral blood (PB) as well as in bone marrow (BM). We therefore became interested in whether endurance exercise has the same effect on very small embryonic-like stem cells (VSELs), which have been described as a population of developmentally early stem cells residing in BM. Mice were run daily for 1 hour on a treadmill for periods of 5 days or 5 weeks. Human volunteers had trained in long-distance running for one year, six times per week. FACS-based analyses and RT-PCR of murine and human VSELs and HSPCs from collected bone marrow and peripheral blood were performed. We observed that endurance exercise increased the number of VSELs circulating in PB and residing in BM. In parallel, we observed an increase in the number of HSPCs. These observations were subsequently confirmed in young athletes, who showed an increase in circulating VSELs and HSPCs after intensive running exercise. We provide for the first time evidence that endurance exercise may have beneficial effects on the expansion of developmentally early stem cells. We hypothesize that these circulating stem cells are involved in repairing minor exercise-related tissue and organ injuries.

## 1. Introduction

Bone marrow (BM) contains a variety of stem cells, including hematopoietic stem/progenitor cells (HSPCs), endothelial progenitor cells (EPCs), mesenchymal stem cells (MSCs), and the dormant population of stem cells from early embryonic development that have been named very small embryonic-like stem cells (VSELs) [1–3]. It has been shown that BM-derived stem cells, especially HSPCs, circulate in peripheral blood (PB) at a very low level under steady-state conditions [4]. This circulation allows the pool of stem cells maintained in BM to be spread to bones located in distant parts of the body. Another important suggested purpose of this circulation is that various types of such circulating stem cells play a role in “patrolling” peripheral tissues to prevent infections and tissue damage [5].

Evidence has also accumulated that HSPCs and EPCs expand in bone marrow (BM) in response to endurance exercise and are subsequently mobilized into peripheral blood (PB) [6–9]. Therefore, we became interested in whether the pool of BM-residing VSELs would respond in a similar way as HSPCs to endurance exercise. These cells, as demonstrated in several reports, have the ability to differentiate into cells from all three germ layers [10] and play an important role in tissue and organ rejuvenation [11], and their number positively correlates with life span in experimental animals [12].

Egress of stem cells from the BM is triggered by activation of the complement cascade, which releases important active cleavage fragments, such as the C5 component C5a, that induce granulocytes and monocytes in BM to release proteolytic enzymes that attenuate stem cell retention signals in BM niches and permeabilize the BM–PB barrier, thus facilitating the egress of stem cells [13]. The major chemoattractant for stem cells in PB is sphingosine-1-phosphate (S1P), and, in the process of mobilization, certain other factors are also involved, including *α*-chemokine stromal-derived factor 1 (SDF-1) [14]. It has also been proposed that mobilization of particular types of stem cells may be modulated by stem cell type-specific factors, such as vascular endothelial growth factor (VEGF), which is involved in mobilization of EPCs [15]. Our recent research demonstrated that mobilization of stem cells not only is triggered by but also correlates with activation of the complement cascade, and its activation can be easily measured in PB using commercially available ELISA assays to detect, for example, the C5b-C9 membrane attack complex (MAC) [16].

Our data confirm that HSPCs are released from BM into PB in response to physical exercise and demonstrate for the first time that, in parallel, VSELs are also mobilized. Importantly, we demonstrate a positive effect of exercise on expansion of this primitive pool of stem cells in BM. Since these small cells may differentiate into several types of cells across the three germ layers, their increase may explain, in a novel way, the positive effect of regular exercise on tissue and organ rejuvenation. Of note, a similar positive effect on this pool of cells by caloric restriction has recently been demonstrated [17]. Therefore, VSELs may be able to reconcile reported observations of a positive effect of both exercise and caloric restriction on life quality and extension of life span. However, we are aware that more direct data are necessary to fully support this novel concept.

## 2. Materials and Methods

### 2.1. Animal Care and Use

Conducted studies were approved by the II Local Ethical Committee, Environmental and Life Science University, and adhered to American College of Sports Medicine (ACSM) animal care standards.

### 2.2. Experimental Animals

The experiments were performed on 90 4-week-old C57BL/6 mice. Sedentary control (SED) and exercise-trained (EX) C57Bl/6 mice were housed three per cage in an ultraclean facility on ventilated racks and were provided food and water* ad libitum* during the experiment period. The animals were purchased from the Animal Laboratory House, Wroclaw Medical School, and housed in the Animal Experimental Laboratory (Wroclaw Medical School, Norwida 34, Poland). Mice were maintained on a 12 h light-dark cycle at 22 ± 0.2°C.

#### 2.2.1. Mice Endurance Exercise on Treadmill

The animals used in this study were divided into two groups: sedentary control animals, which did not undergo physical activity (*n* = 6), and animals undergoing physical activity (*n* = 6). Animals were exercise-trained (*n* = 6) on an Exer 3/6 Treadmill (Columbus Instruments, Columbus, OH, USA) 3 d/wk (Monday, Wednesday, and Friday) for 5 wk. The mice were accustomed to the treadmill a week before training. For the 5 wk training period, mice were subjected to a progressive exercise protocol, with the training portion of the protocol beginning at 14 m/min for 45 min (wk 1) and increasing to 24 m/min for 45 min (wk 5). The training portion of the protocol was always preceded by a 10 min warm-up at 10 m/min and followed by a 5 min cool-down at 10 m/min as described previously [8]. SED mice (*n* = 6) were exposed to the treadmill and were given similar inducements on the same days as EX mice but were not subjected to training. The training intensity corresponded to 70–75% of VO_2max_ (murine maximal oxygen uptake). Electrical stimulation was not used to encourage the animals to run.

#### 2.2.2. Mice Exercise on Rotating Wheels

Twelve 4-week-old C57BL/6 mice were accustomed for 7 days to the presence of a rotating wheel and were subsequently subjected to controlled 45-minute exercise on rotating wheels. Standard mouse exercise wheels were attached directly to the side wall of the cages, and each wheel had a magnet directly attached. Prior to commencement of the running trial, the mice in the EX group were placed in a cage containing an exercise wheel and kept there for 7 days.

#### 2.2.3. Human Volunteers

Twelve healthy volunteers, with a mean age of 24 ± 1 years (20–28 years old), mean body weight of 85.0 ± 3.2 kg (68.0–93.5 kg), and mean VO_2max_ of 49.4 ± 1.6 mL kg^−1 ^min^−1^ (43.3–55.1 mL, kg^−1^, min^−1^) were recruited to take part in the experiment. All individuals were divided into two groups: sedentary (*n* = 6) and physically active students (*n* = 6). The volunteers recruited to the sedentary group were students at Wroclaw Medical School and were not engaged in regular training. Physically active students were recruited from Wroclaw Sport Club (WKS Slask) and had trained in long-distance running for one year, with endurance training performed six times per week during the year. The regular training sessions consisted of endurance running at a distance of 9-10 km/day with a speed of 0.2 km/min, which is equivalent to ~65% of VO_2max_. The peripheral blood from physically active students was collected 30 min after the last training session in the Faculty of Pharmacy, Medical University in Wroclaw. Following routine medical screening, subjects were advised of the purpose of the study and associated risks, and all provided written informed consent. The experimental protocol was approved by the Local Ethical Committee at Wroclaw Medical School.

#### 2.2.4. FACS-Based Analysis of VSELs and HSPCs from Murine BM and PB

Total nucleated cells (TNCs), which were obtained from BM and PB, were subsequently stained for CD45, hematopoietic lineage markers (Lin), and Sca-1 antigen for 30 min in medium containing 2% fetal bovine serum. The following anti-mouse antibodies (BD Pharmingen) were used for staining: rat anti-CD45 (allophycocyanin/Cy7, clone 30F11), anti-CD45R/B220 (PE, clone RA-6B2), anti-Gr-1 (PE, clone RB6-8 C5), anti-T-cell receptor-*αβ* (PE, clone H57-5970), anti-T-cell receptor-*ɤδ* (PE, clone GL3), anti-CD11b (PE, clone M1/70), anti-Ter119 (PE, clone TER-119), and anti-Ly-6A/E (also known as Sca-1, biotin, clone E13-161.7, with streptavidin conjugated to PE–Cy5). Cells were then washed, resuspended in RPMI medium with 2% fetal bovine serum, and sorted with an Influx cell sorter (BD, CA, USA). Two populations were analyzed: Lin^−^Sca-1^+^CD45^−^ (VSELs) and Lin^−^Sca-1^+^CD45^+^ (HSPCs) [18].

#### 2.2.5. FACS-Based Analysis of VSELs and HSPCs from Human PB

Whole human PB was lysed in BD lysing buffer (BD Biosciences, USA) for 15 min at room temperature and washed twice in phosphate-buffered saline (PBS). A single-cell suspension was stained for lineage markers (CD2 clone RPA-2.10, CD3 clone UCHT1, CD14 clone M5E2, CD66b clone G10F5, CD24 clone ML5, CD56 clone NCAM16.2, CD16 clone 3G8, CD19 clone HIB19, and CD235a clone GA-R2) conjugated with fluorescein isothiocyanate (FITC), CD45 (clone HI30) conjugated with phycoerythrin (PE), and a combination of CD133 (CD133/1) conjugated with APC, CD34 (clone 581) conjugated with PB, CD31 (clone WM59) (APC-Cy7), and CD51 (clone 23C6 RUO) conjugated with PE-Cy7 for 30 min on ice. After washing, VSELs (CD45^−^Lin^−^CD133^+^ and CD45^−^Lin^−^CD34^+^ cells), HSPCs (CD45^+^Lin^−^CD133^+^ and CD45^+^Lin^−^CD34^+^ cells), EPCs (CD34^+^CD133^+^KDR^+^), and MSCs (CD45^−^Lin^−^CD31^−^ CD51^+^) were analyzed by fluorescence-activated cell sorting (FACS, Navios, Beckman Coulter, USA). At least 10^6^ events were acquired and analyzed using Kaluza software.

#### 2.2.6. Real-Time Quantitative Reverse Transcription PCR (RQ-PCR)

Total RNA was isolated from cells harvested from BM and PB from experimental and control mice by employing the RNeasy Kit (Qiagen, Valencia, CA). The RNA was reverse-transcribed with MultiScribe reverse transcriptase and oligo-dT primers (Applied Biosystems, Foster City, CA). Quantitative assessment of mRNA levels was performed by real-time reverse transcriptase polymerase chain reaction (RT-PCR) on an ABI 7500 Fast instrument employing Power SyBR Green PCR Master Mix reagent. Real-time conditions were as follows: 95°C (15 sec) followed by 40 cycles at 95°C (15 sec) and 60°C (1 min). According to melting point analysis, only one PCR product was amplified under these conditions. The relative quantity of target, normalized to the endogenous control *β*-2 microglobulin gene and relative to a calibrator, is expressed as fold change (2^−ΔΔCt^), where ΔCt = (Ct of target gene) − (Ct of the endogenous control gene, *β*-2 microglobulin) and ΔΔCt = (ΔCt of target gene) − (ΔCt of calibrator for the target gene). The following primer pairs were used: *β*2-microglobin, 5′-CAT ACG CCT GCA GAG TTA AGC A-3′ (forward) and 5′-GAT CAC ATG TCT CGA TCC CAG TAG-3′ (reverse); Oct-4, 5′-TTC TCA ATG CTA GTT CGC TTT CTC T-3′ (forward) and 5′-ACC TTC AGG AGA TAT GCA AAT CG-3′ (reverse); Sox2, 5′-GCG GAG TGG AAA CTT TTG TCC-3′ (forward) and 5′-GGG AAG CGT GTA CTT ATC CTT CT-3′ (reverse); Rex1, 5′-AGA TGG CTT CCC TGA CGG ATA-3′ (forward) and 5′-CCT CCA AGC TTT CGA AGG ATT T-3′ (reverse).

#### 2.2.7. *In Vitro* Clonogenic Assays of Murine HSPCs

The growth of murine HSPCs isolated from BM and PB was evaluated by an* in vitro* clonogenic assay, as described previously [17]. Briefly, 2 × 10^5^ BM-derived and 4 × 10^5^ PB-derived cells were resuspended in 0.4 mL of RPMI-1640 medium and mixed with 1.8 mL of MethoCult HCC-4230 methylcellulose medium (StemCell Technologies Inc., Canada), supplemented with L-glutamine and antibiotics. Specific murine recombinant growth factors (all from R&D Systems, USA) were added. To stimulate granulocyte-macrophage colony-forming units (CFU-GM), IL-3 (20 U/mL), SCF (10 ng/mL), and GM-CSF (5 ng/mL) were used. EPO (5 U/mL), SCF (10 ng/mL), and IL-3 (20 U/mL) were used to stimulate erythrocyte burst-forming units (BFU-E). The colonies were counted under an inverted microscope after 7–10 days of culture. Each clonogenic assay was performed in quadruplicate.

#### 2.2.8. ELISA to Detect Murine MAC, SDF-1, and VEGF

Fifty microliters of PB was taken from the vena cava of the six C57BL/6 mice and collected into Microvette EDTA-coated tubes (Sarstedt Inc., Newton, NC). The concentration of C5b-C9 (MAC) was measured by employing the commercially available, highly sensitive enzyme-linked immunosorbent assay (ELISA) kit K-ASSAY (Kamiya Biomedical Company, Seattle, WA, USA), according to the manufacturer's protocol. The concentrations of SDF-1 and VEGF were measured by employing the commercially available, highly sensitive enzyme-linked immunosorbent assay (ELISA) (R&D Systems, Europe Ltd.).

#### 2.2.9. ELISA to Detect Human Oct-4, Sox2, and Nanog

Fifty microliters of PB was taken from the vena cava of the six C57BL/6 mice and collected into Microvette EDTA-coated tubes (Sarstedt Inc., Newton, NC). The concentrations of Oct-4, Sox2, and Nanog were measured by employing the commercially available, highly sensitive enzyme-linked immunosorbent assay (ELISA) (MyBioSource), according to the manufacturer's protocol. Results were calculated and presented as percentage fold difference.

#### 2.2.10. Statistical Analysis

All data were analyzed using Microsoft Excel 2007 and Statistica version 7.1 software. Most results are presented as mean ± standard error of the mean. The Mann-Whitney *U* and Student's *t*-tests were used, and statistical significance was defined as *P* < 0.05.

## 3. Results

### 3.1. Short Exercise on Rotating Wheels Mobilizes VSELs into Peripheral Blood

**Figure 1:**
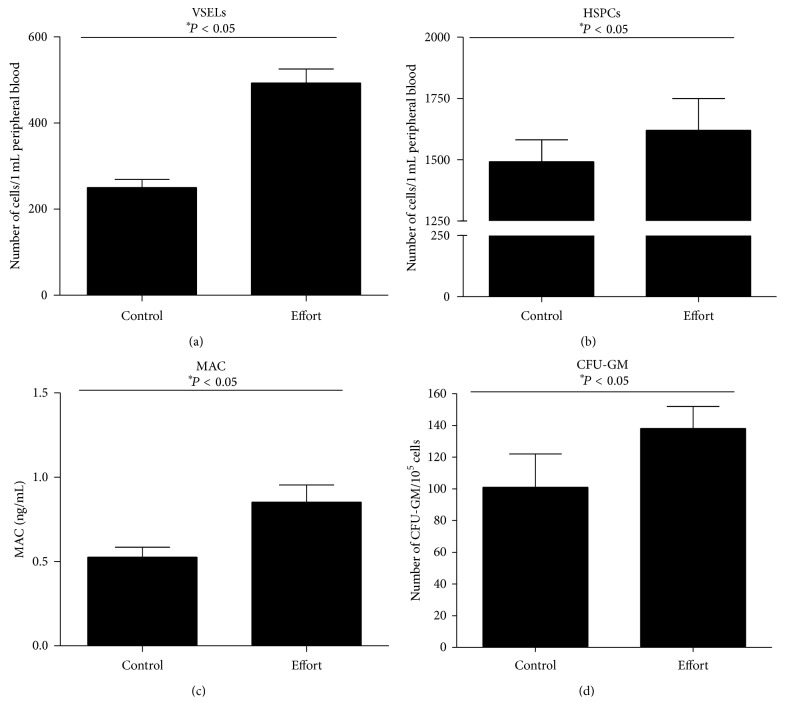
Effect of short (45 min) exercise on a rotating wheel on stem cell mobilization in mice. (a, b) The number of VSELs (a) and HSPCs (b) circulating in PB after 45 min of exercise on a rotating wheel (effort) compared with nonexercising mice (control). (c) Activation of the complement cascade in PB after 45 min of exercise on a rotating wheel (effort) compared with nonexercising mice (control) measured by C5b-C9 (MAC) ELISA. (d) The increase in the number of clonogenic progenitors circulating in PB after 45 min of exercise on rotating wheels (effort) compared with nonexercising mice (control) (*n* = 6 mice/group).

It has been demonstrated that physical activity mobilizes HSPCs, both in mice and humans [6–9]. To address the effect of short periods of physical activity on the mobilization of VSELs, we exercised normal mice for 45 minutes on a standard rotating wheel, and immediately afterwards mice were evaluated for the numbers of VSELs and HSPCs circulating in peripheral blood (PB, Figures 1(a) and 1(b)) and activation of the complement cascade (Figure 1(c)), which, as we demonstrated previously, is crucial for egress of stem cells from the bone marrow (BM) microenvironment into PB [13–15]. We observed a significant ~2x increase in the numbers of VSELs (Figure 1(a)), increased numbers of HSPCs (Figure 1(b)), and increased numbers of clonogenic CFU-GM circulating in PB (Figure 1(d)). The increases in number of these circulating cells correlated with activation of the complement cascade, as evidenced by an increase in C5bC9 (also known as the membrane attack complex (MAC)) measured in PB plasma by ELISA (Figure 1(c)).

### 3.2. Prolonged Endurance Exercise on a Treadmill Increases the Number of VSELs Circulating in PB as well as Residing in BM

**Figure 2:**
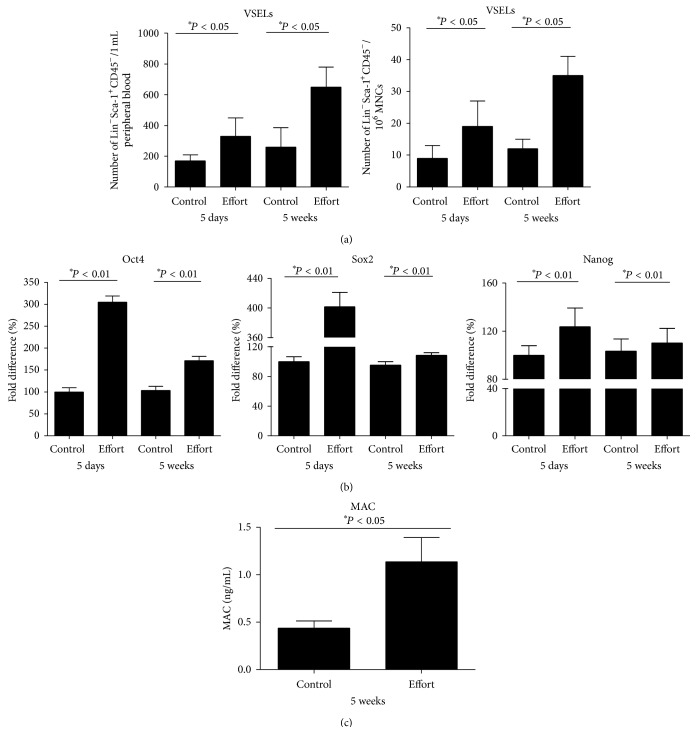
Effect of 5 days or 5 weeks of exercise on a treadmill on the number of VSELs in PB and BM. (a) The number of VSELs circulating in PB (left panel) and BM (right panel) after 5 days or 5 weeks of exercise on a treadmill (effort) compared with nonexercising mice (control). (b) Quantitative real-time PCR changes in expression of Oct4, Sox2, and Nanog in PB mononuclear cells after 5 days or 5 weeks of exercise on a treadmill (effort) compared with nonexercising mice (control), whose expression level was defined as 100%. (c) Activation of the complement cascade in PB measured by C5b-C9 (MAC) ELISA after 5-week exercise on a treadmill (effort) compared with nonexercising mice (control) (*n* = 6 mice/group).

Subsequently, we performed an endurance exercise experiment in which mice were subjected to forced running on a treadmill for 5 days or 5 weeks.  Figure 2(a) shows that forced exercise for 5 consecutive days or 5 weeks significantly enhanced the number of VSELs both circulating in PB (left panel) and residing in BM (right panel). This increase in the number of VSELs circulating in PB correlated with an increase in expression of mRNA for VSEL markers such as Oct-4, Sox2, and Nanog [11, 12] in PB mononuclear cells (Figure 2(b)). Again, mobilization of VSELs correlated with an increase in activation of the complement cascade (Figure 2(c)). A representative FACS analysis of VSELs circulating in PB in control mice as well as mice that had exercised for 5 days or 5 weeks is shown in* Supplemental Digital Content* available online at http://dx.doi.org/10.1155/2016/5756901.

Interestingly, these changes in the numbers of VSELs and HSPCs did not correlate with the plasma levels of SDF-1 or VEGF. In fact, we did not observe significant changes in the levels of these factors in PB between control and exercising mice (data not shown), which indicates, along with our previous observations in other models of stem cell mobilization, that if the complement cascade is activated and stem cells are released from their niches in BM due to a proteolytic microenvironment, the plasma level of S1P is sufficiently high to induce egress of stem cells from BM into PB [14].

**Figure 3:**
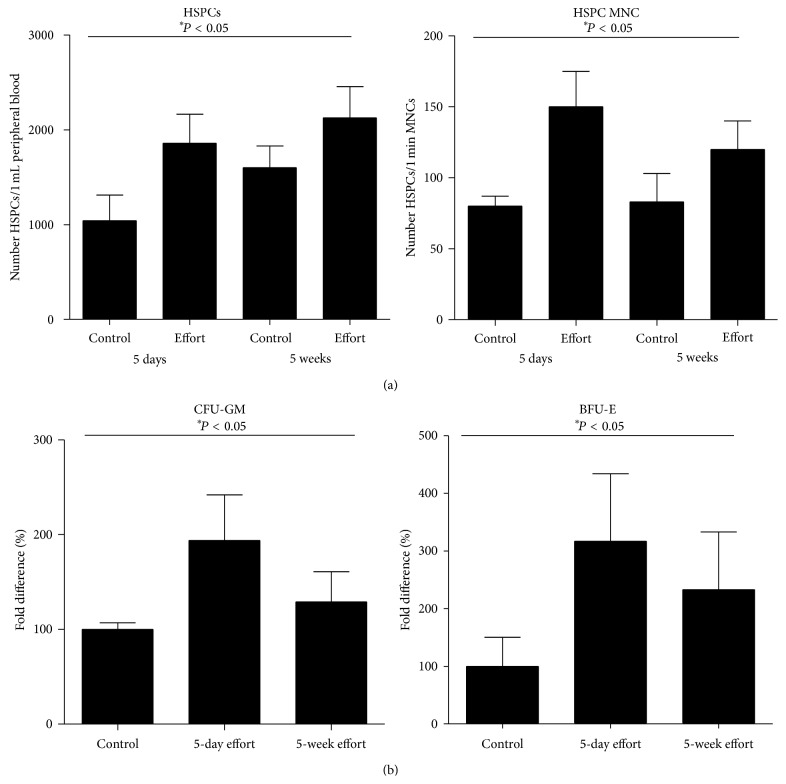
Effect of 5 days or 5 weeks of exercise on a treadmill on the number of HSPCs in PB and BM. (a) The number of HSPCs circulating in PB (left panel) and BM (right panel) after 5 days or 5 weeks of exercise on a treadmill (effort) compared with nonexercising mice (control). (b) The increase in the number of clonogenic progenitors (CFU-GM, left panel, and BFU-E, right panel) in BM after 5 days or 5 weeks of exercise on a treadmill (effort) compared with nonexercising mice (control) (*n* = 6 mice/group).

At the same time, we observed the predicted increase in HSPCs (Figure 3(a)), both circulating in PB (left panel) and residing in BM (right panel). An increase in the numbers of HSPCs in BM correlated with increases in the numbers of clonogenic CFU-GM and BFU-E progenitors (Figure 3(b)).

### 3.3. Increases in VSELs Circulating in PB after Running Exercise in Young Athletes

**Figure 4:**
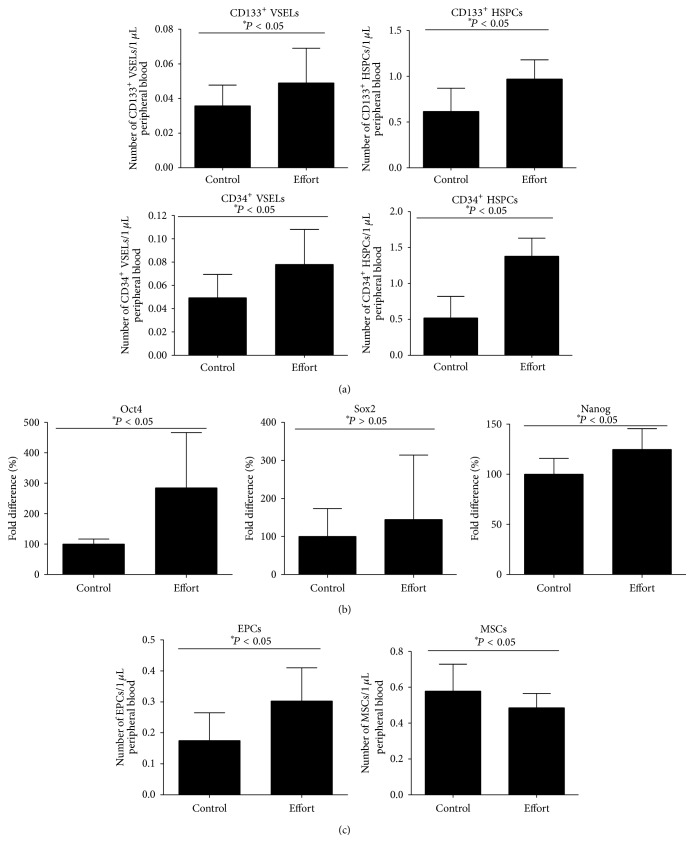
Effect of exercise on stem cell mobilization in healthy young volunteers. (a) The number of CD133^+^ and CD34^+^ VSELs (left) and CD133^+^ and CD34^+^ HSPCs (right) circulating in PB in young athletes after not exercising (control) or after running exercise (effort). (b) Quantitative real-time PCR changes in expression of Oct4, Sox2, and Nanog in PB mononuclear cells after running exercise (effort) compared with nonexercising subjects (control). Changes are shown compared with values detected in control volunteers (defined as 100%). (c) The number of EPCs (left panel) and MSCs (right panel) circulating in PB in young athletes after not exercising (control) or after running exercise (effort) (*n* = 6 volunteers/group).

The effect of running exercise on increasing the numbers of HSPCs circulating in PB has already been demonstrated in humans [8]. In this study, encouraged by our murine data, we enumerated the number of small CD34^+^Lin^−^CD45^−^ and CD133^+^Lin^−^CD45^−^ cells circulating in PB (Figure 4(a)) that correspond to the VSEL population (left panels) in sedentary and physically active students, and, in parallel, we enumerated the number of CD34^+^Lin^−^CD45^+^ and CD133^+^Lin^−^CD45^+^ cells that correspond to HSPCs (right panels). Figure 4(a) shows an increase in the numbers of these cells circulating in PB in physically active subjects after running effort (90–120 min of training session corresponding to 65% of training consisting of VO_2peak_ at the distance of 8 km). An increase in the number of VSELs circulating in PB has been subsequently confirmed by ELISA to detect Oct-4, Sox2, and Nanog proteins (Figure 4(b)). We also observed a small increase in the number of EPCs but not MSCs circulating in PB (Figure 4(c)).

## 4. Discussion

The salient observation of this work is the positive effect of exercise on mobilization into PB and expansion in BM of VSELs. Since these small developmentally early cells may differentiate into several types of cells across all three germ layers and play a role in regeneration [10–12, 18], this observation may explain, in a novel way, the positive effect of regular exercise on tissue and organ rejuvenation.

In addition to VSELs, we have confirmed that physical activity also mobilizes HSPCs into PB. In support of this observation, there are several reports in the literature demonstrating that physical exercise mobilizes both HSPCs and EPCs into PB [6, 8, 9]. The efficacy of the mobilization may vary, depending on the intensity of the exercise protocols employed in human or animal subjects and the time of sample collection of PB or BM after exercise for enumeration of the stem cells. In our experiments, we evaluated the effect of exercise on the release of VSELs and HSPCs from BM immediately after a short, intensive run on rotating wheels or after repeated daily running exercise on a treadmill for 5 days or 5 weeks. We also evaluated the mobilization of VSELs and HSPCs in healthy young athletes after a 10 km run. It is most likely that stem cells mobilized during strenuous exercise act as “circulating paramedics,” with a role in repairing microscopic damage in skeletal muscles as well as in other tissues. An important role in this phenomenon may be played by VSELs, which, in appropriate* in vivo* models, are reportedly able to differentiate into cells for different tissues, including mesenchymal precursors, lung alveolar epithelium, gametes, and endothelial cells [19–22].* In vitro* research proved that not only does an enabling environment improve releasing and increasing cells' proliferation but both mechanical and physical stress, as well changed conditions (i.e., different culture surfaces), are associated with mechanisms controlling cellular functions. These findings provide evidence that endurance training may play modulating role in regulation of cellular functions and VSELs releasing [23–25]. This ability corresponds with several observations in which VSELs are released into PB in clinical situations of organ or tissue damage, such as heart infarct [26], stroke [27], skin burns [28], and neural tissue toxic damage [29], and the extent of their mobilization into PB may even have some prognostic value [26–28]. Our data presented herein show for the first time that not only are VSELs released from BM into PB in response to physical exercise but there is also a positive effect of exercise on the expansion of this primitive pool of stem cells in BM.

We also observed that endurance exercise increases the number of HSPCs in BM, corroborating reports from other investigators [7, 9]. Specifically, mice trained on a treadmill at progressive speeds over a 10-week period displayed increased medullary and mobilized HSPC content from 50 to 800%, depending on the HSPC type, and marrow cavity fat was reduced by 78% [6]. Interestingly, as has been demonstrated in another paper from this group, exercise promoted BM cell survival, and exercise training increased survival of recipient mice after BM transplantation with increased total blood cell reconstitution [30]. Based on this finding, endurance training has a positive prohematopoietic effect, both directly on HSPCs and on accessory cells in the BM microenvironment (e.g., stroma and osteoblasts) that provide niches for these cells [4]. Interestingly, in addition to HSPCs, it has been reported that physical activity also has a positive effect on neurogenesis in the adult and aging brain [31, 32]. Thus, physical activity has a positive effect on several types of tissue-committed stem cells in various organs, such as skeletal muscle satellite stem cells [31, 33], HSPCs [9], and neural progenitors, and several factors triggered by exercise, such as insulin-like growth factor 1, VEGF, platelet-derived growth factor, hepatocyte growth factor, and hormones such as androgens, mediate this effect [31]. On the other hand, it is very likely that other non-peptide-based regulators of cell growth, such as bioactive phosphosphingolipids and alarmines released from hypoxic tissues, may play an important role as well [31].

Mobilization into PB and expansion in BM of VSELs, which may differentiate into several types of cells across the three germ layers [10, 11, 19–22] and express several genes characteristic of early development stem cells (Oct-4, Sox-2, and Nanog), may explain, in a novel way, the positive effect of regular exercise on tissue and organ rejuvenation. Of note, there is a similar positive effect on this pool of stem cells by caloric restriction, as we demonstrated recently [16]. Therefore, VSELs may reconcile all observations reported so far of a positive effect of both exercise and caloric restriction on life quality and the extension of life span [12]. However, we are aware that more direct data are necessary to fully support this novel concept.

Mobilization of both HSPCs and VSELs correlated in our studies with activation of the complement cascade. This again confirms the pivotal role of this cascade, which is also seen as a response to infection or tissue and organ damage in triggering the mobilization process after strenuous exercise [13]. Since the complement cascade has robust cross-talk with two other evolutionarily ancient proteolytic cascades—the coagulation and fibrinolytic cascades [34]—further studies are needed to assess the role of the products of activation of these cascades on mobilization of stem cells in response to endurance exercise.

We conclude that physical activity may have a positive effect on improving life quality by directly affecting the pool of the most primitive stem cells residing in adult tissues. Future studies will be important to address the question of whether a combination of physical activity with caloric restriction and some pharmacological drugs, such as metformin, which is currently employed to increase life span, have a synergistic effect on VSELs and tissue and organ rejuvenation. These observations will be crucial for the development and optimization of novel treatment strategies aimed at prolonging human life span, and physical activity is an important part of this.

## Supplementary Material

A representative FACS analysis of VSELs circulating in PB in control mice as well as mice that had exercised for 5 days or 5 weeks.
