# Supplementary material for: Endurance Exercise Mobilizes Developmentally Early Stem Cells into Peripheral Blood and Increases Their Number in Bone Marrow: Implications for Tissue Regeneration
Source: Stem Cells Int. 2015 Nov 9;2016:5756901. doi: 10.1155/2016/5756901 (PMC4655293; doi:10.1155/2016/5756901)
Supplement: Supplementary file 1 — A representative FACS analysis of VSELs circulating in PB in control mice as well as mice that had exercised for 5 days or 5 weeks. [file 5756901.f1.pdf]

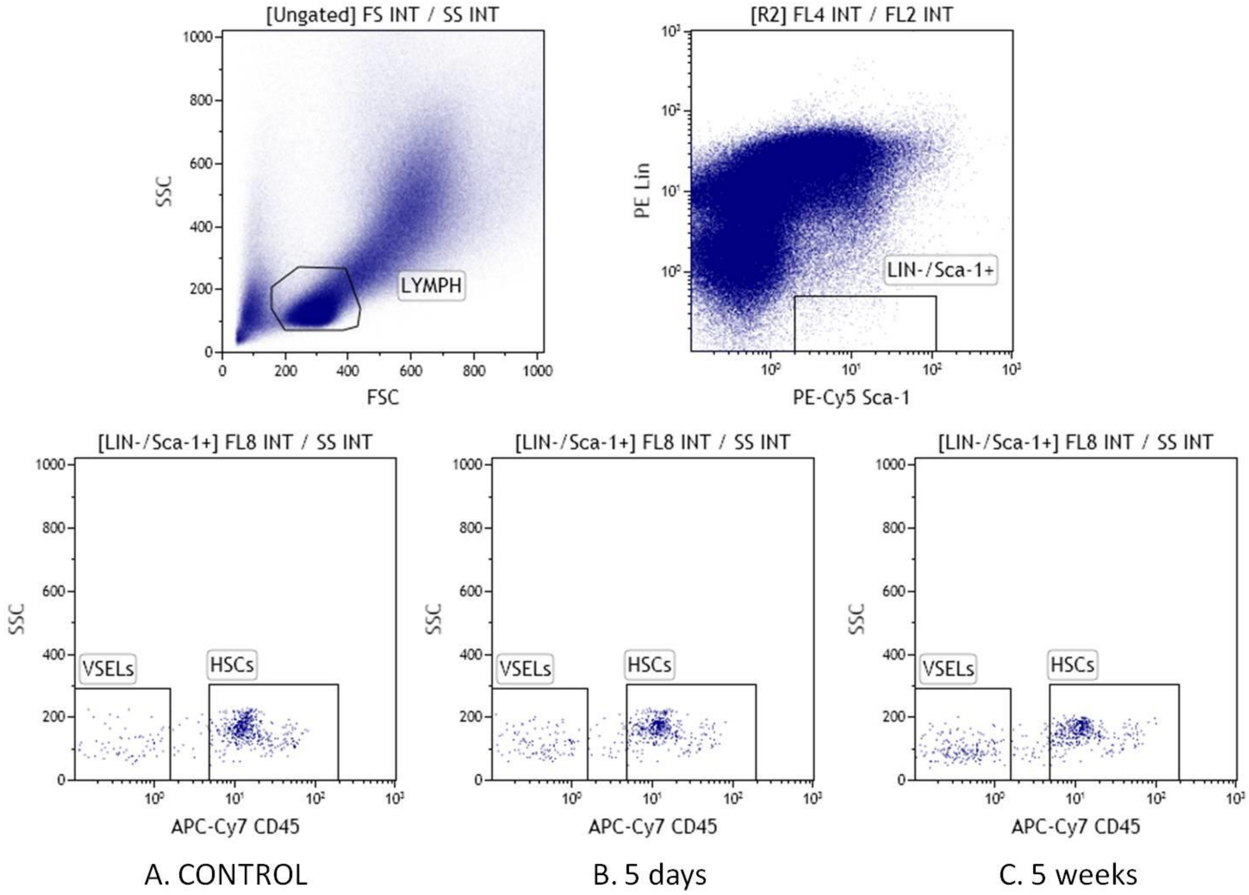


**Supplementary Figure 1** Flow cytometry analysis of the VSELs and HPSCs circulating in PB in control mice after their mobilization into PB. Upper panel, representative gating strategy. Lower panel, the number of VSELs and HSPCs circulating in PB in control mice and mice exercising on a treadmill for 5 days or 5 weeks. Representative data are shown.
